# Weight change across adulthood in relation to ultrasound-defined metabolic dysfunction-associated steatotic liver disease: a population-based study

**DOI:** 10.7189/jogh.16.04075

**Published:** 2026-03-20

**Authors:** Xian Cui, Jing Shi, Darong Hao, Chunbei Yi, Ziyu Zhang, Cui Wu, Yanyan Yang, Yanting Zhang, Jun Du, Fangfang Xu, Xiangwei Li

**Affiliations:** 1 Diagnostic Imaging Center, Shanghai Children's Medical Center, Shanghai Jiao Tong University School of Medicine, Shanghai, China; 2 School of Global Health, Chinese Centre for Tropical Diseases Research, Shanghai Jiao Tong University School of Medicine, Shanghai, China; 3 Shanghai Baoshan District Center for Disease Control and Prevention, Shanghai, China; 4 Xinyang Center for Disease Control and Prevention, Henan, China; 5 Hainan International Medical Center, Shanghai Jiao Tong University School of Medicine, Hainan, China; 6 Department of Geriatric Medicine, Henan Provincial People's Hospital, Henan, China

## Abstract

**Background:**

Although obesity is a well-established risk factor for metabolic dysfunction-associated steatotic liver disease (MASLD), the impact of long-term weight trajectories on MASLD risk remains unclear. We aimed to examine the association between weight change trajectories across adulthood and the risk of ultrasound-defined MASLD.

**Methods:**

We analysed data from 4999 participants from the Healthy Happy Aging Community Cohort. Weight trajectories were assessed using body mass index (BMI) at age 25 years, ten years before baseline, one year before baseline, and at baseline. MASLD was defined as a median (MD) controlled attenuation parameter (CAP) of ≥274 dB/m and severe steatosis as a MD CAP of ≥302 dB/m via vibration-controlled transient elastography.

**Results:**

There were 2247 (44.9%) patients with MASLD, of whom 1468 (29.4%) had severe steatosis. Compared with stable normal weight, sustained obesity (BMI ≥ 30 kg/m^2^ at all time points) demonstrated a strong association with MASLD risk (adjusted odds ratio (aOR) = 2.17; 95% confidence interval (CI) = 1.64–2.86), particularly for severe steatosis (aOR = 2.52; 95% CI = 1.74–3.66). The transition from non-obese to obese status was associated with a 2.5-fold increase in risk (aOR = 2.51; 95% CI = 1.81–3.48), with a more pronounced effect observed in men (*P*-interaction <0.05). Weight gain of ≥5 kg between ages 25–40 years showed particularly strong associations (aOR = 2.20; 95% CI = 1.70–2.86), while baseline severe obesity (BMI ≥ 35 kg/m^2^) remained the strongest independent predictor (aOR = 4.12; 95% CI = 2.70–6.29).

**Conclusions:**

Long-term obesity and weight gain, especially in early adulthood, significantly increase MASLD risk. Sustained obesity and progression to obesity were key risk factors, with severe obesity showing the strongest association. Early weight management is crucial for MASLD prevention.

Metabolic dysfunction-associated steatotic liver disease (MASLD) has emerged as a global health challenge and is characterised by excessive hepatic fat accumulation (>5% hepatocytes) unrelated to alcohol consumption or other identifiable causes. Affecting approximately 25% of the global population [1,2], MASLD encompasses a disease spectrum ranging from simple steatosis to non-alcoholic steatohepatitis, ultimately leading to severe liver-related morbidity (including cirrhosis, end-stage liver disease, and hepatocellular carcinoma) and increased mortality [3,4]. This substantial disease burden underscores the urgent need for further research on risk factors.

The pathogenesis of MASLD follows the ‘multiple hits hypothesis’, which involves complex interactions between genetic predisposition and environmental factors [5]. Among these, obesity is the principal driver, promoting both the initial development of hepatic steatosis and subsequent progression to non-alcoholic steatohepatitis [6]. The parallel increase in MASLD prevalence and global obesity rates strongly suggests a pathophysiological link [6]. However, current research remains limited by its reliance on single-time point body mass index (BMI) measurements, which fail to account for the dynamic nature of weight fluctuations across the lifespan [6,7]. This methodological gap highlights the need for longitudinal assessments of weight-trajectory patterns.

Emerging evidence from mortality studies has demonstrated that weight change patterns, including sustained obesity, young-to-middle adulthood weight gain, and middle-to-late adulthood weight loss, have distinct prognostic implications [8]. Crucially, these studies establish that dynamic BMI assessment outperforms static measurements in risk prediction [8]. Despite these advances, the relationship between long-term weight trajectories and MASLD risk remains unexplored, which represents a significant knowledge gap in hepatology research.

While current BMI remains a strong determinant of MASLD, it remains unclear whether long-term weight trajectories provide additional risk stratification among individuals with similar contemporary BMI. To address this gap, we analysed data from the Healthy Happy Aging Community Cohort, employing transient elastography, a validated, non-invasive modality with established accuracy in MASLD screening [9], to examine the relationship between weight changes from young adulthood (25 years) to midlife and late adulthood with MASLD.

## METHODS

### Study population

The Healthy Happy Aging Community Cohort study is a longitudinal, community-based cohort established to monitor health status and disease progression among older adults in China. Participants were recruited from three community health service centres located in Henan Province (Central China) and Jiangsu Province (Eastern China). The health examination programme commenced in 2015 and included annual follow-up assessments. All participants completed standardised in-person interviews, physical examinations, and laboratory assessments performed by physicians who had undergone unified professional training.

Structured face-to-face questionnaires were administered to collect sociodemographic information, lifestyle behaviours (*i.e.* smoking, alcohol intake, and physical activity), medical history, medication use, and detailed weight history. Anthropometric measurements, including height and body weight, were obtained following standardised protocols, with participants wearing light clothing and no shoes. Controlled attenuation parameter (CAP) values and liver stiffness measurements were obtained using vibration-controlled transient elastography operated by trained personnel.

**Figure 1:**
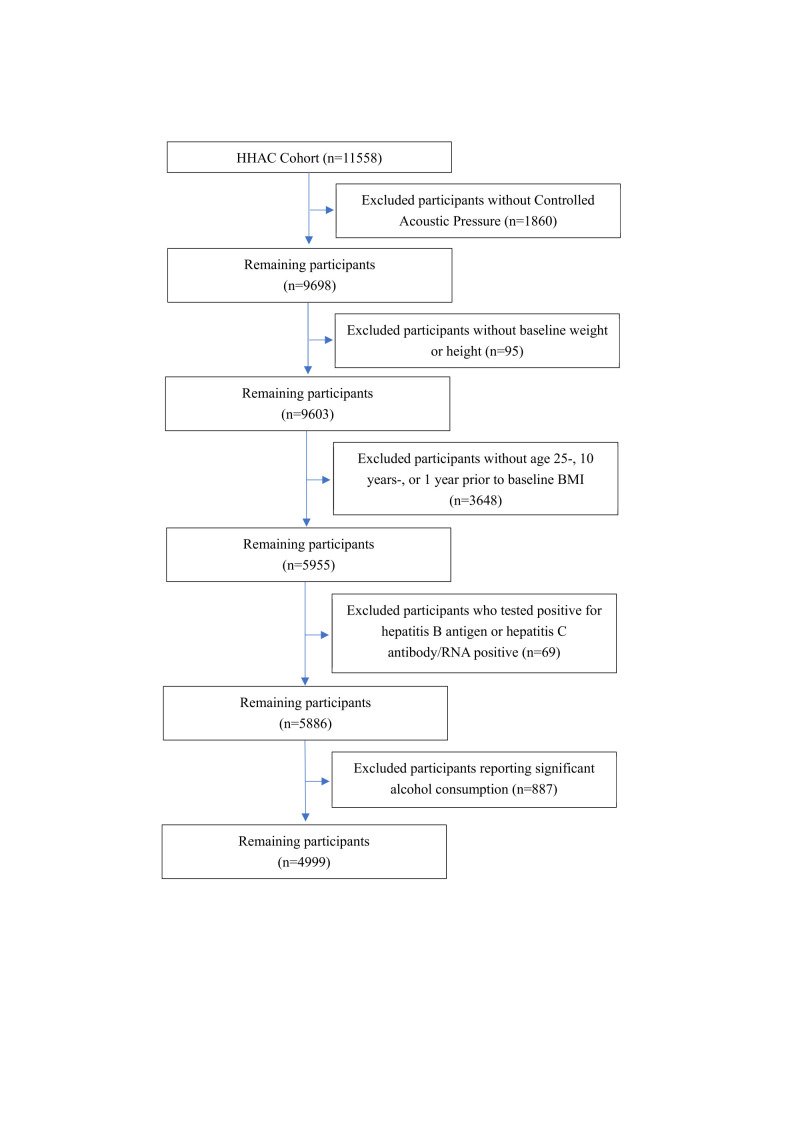
Flowchart of participant selection from the HHAC cohort. BMI – body mass index, HHAC – Healthy Happy Aging Community.

A total of 11 558 individuals participated in the initial health examinations (**Figure 1**). We excluded participants who lacked CAP measurements (n = 1860), baseline anthropometric data (n = 95), or BMI records at age 25 years, ten years before baseline, or one year before baseline (n = 3648). We also excluded individuals who tested positive for hepatitis B surface antigen or hepatitis C antibody/RNA (n = 69) and those reporting significant alcohol consumption (≥4 drinks/d for women and ≥5 drinks/d for men) (n = 887). The final analytic cohort comprised 4999 participants. We reported this study in accordance with the STROBE guideline (Table S1 in the **Online Supplementary Document**) [10].

### Assessments of weight change

Baseline was defined as the time point at which participants underwent physical examinations. Body weight at three historical time points (age 25 years, ten years before baseline, and one year before baseline) was self-reported during the survey, whereas baseline weight and height were measured during standardised physical examinations. Self-reported historical weight has demonstrated reasonable validity and reproducibility in adult populations, including older adults, and is widely used in life-course epidemiologic studies. Any non-differential misclassification would be expected to bias associations toward the null. BMI was calculated as weight (kg) divided by height squared (m^2^) at four time points: age 25 years, ten years before baseline, one year before baseline, and at baseline. Participants were categorised into three BMI groups (<25.0, 25.0–29.9, and ≥30.0 kg/m^2^) at each time point. These thresholds were selected based on World Health Organization criteria and prior life-course studies to enhance comparability and clinical interpretability.

Following previously established methodologies [8,11], we analysed weight change patterns across five intervals: at age 25 to ten years prior to baseline, ten years prior to baseline to one year prior to baseline, at age 25 to baseline, ten years prior to baseline to baseline, and one year prior to baseline to baseline. Patterns were classified as stable normal (<25.0 both times), maximum overweight (25.0–29.9 at either time), obese (≥30.0) to non-obese (<30.0), non-obese (<30.0) to obese (≥30.0), or stable obese (≥30.0 both times).

### Ascertainment of MASLD

Hepatic steatosis was assessed using median (MD) CAP scores, measured via vibration-controlled transient elastography performed by trained technicians using the FibroScan® 502 V2 Touch device. Consistent with the established diagnostic criteria, MASLD was defined as an MD CAP value of ≥274 dB/m, a threshold demonstrating 90% sensitivity for detecting hepatic steatosis of any severity [12]. To further characterise disease severity, we applied a secondary cut-off (MD CAP≥302 dB/m) to identify participants with severe steatosis, as validated in prior studies [13,14].

### Assessment of covariates

We also included demographic, lifestyle, and clinical covariates in the analysis. Demographic characteristics included age at baseline, sex, ethnicity (Han and ethnic minorities), education level (less than high school, high school graduate, and college or above), and place of residence. Lifestyle factors included smoking status (categorised as never or ever smoker) and participation in moderate-intensity physical activity (yes/no), obtained through standardised questionnaires.

Trained health technicians collected anthropometric measurements, including standing height, body weight, and waist circumference, using standardised protocols. Height was measured to the nearest 0.1 cm with a stadiometer, weight to the nearest 0.1 kg on a digital scale, and waist circumference at the iliac crest to the nearest 0.1 cm, with participants wearing light examination gowns and no shoes.

Clinical laboratory measurements were performed in certified clinical laboratories affiliated with the participating community health service centres. All biochemical indicators were analysed using standardised automated analysers following manufacturers’ protocols and routine internal quality-control procedures. Serum total cholesterol and high-density lipoprotein cholesterol were measured using enzymatic assays widely employed in clinical practice. Liver function indicators, including alanine aminotransferase, aspartate aminotransferase, and alkaline phosphatase, were quantified using automated biochemical analysers with regular calibration and external quality assessment. All laboratory procedures adhered to national clinical laboratory standards, and quality-control materials were used throughout the testing process to ensure accuracy and reliability.

### Statistical analysis

We summarised the demographic characteristics of the study participants using standard descriptive methods. We assessed correlations between BMI at four time points and weight changes across five intervals using Pearson correlation coefficients (r), with scatter plots of absolute BMI differences *vs.* CA*P* values.

We used multivariable logistic regression models to estimate odds ratios (ORs) and 95% confidence intervals (CIs) for associations between MASLD and BMI at each time point, and to examine weight-change patterns across the four time intervals. We examined each BMI time point and weight-change interval in separate models to avoid collinearity among highly correlated anthropometric measures. All analyses were hypothesis-driven and based on pre-specified exposure definitions. We conducted separate analyses for MASLD (MD CAP≥274 dB/m) and severe steatosis (MD CAP≥302 dB/m). We initially adjusted the models for age, sex, and study site (Model 1), and then further adjusted for educational level, place of residence, moderate physical activity, smoking status, waist circumference, high-density lipoprotein cholesterol, alanine aminotransferase, aspartate aminotransferase, and alkaline phosphatase (Model 2).

We first examined the associations between BMI at each time point and MASLD risk by categorising BMI into seven groups: underweight (<18.5 kg/m^2^), three normal weight categories (18.5–19.9, 20.0–22.4, and 22.5–24.9 kg/m^2^), overweight (25.0–29.9 kg/m^2^), and two obesity categories (30.0–34.9 and ≥35.0 kg/m^2^), using the 22.5–24.9 kg/m^2^ group as the reference. In our primary analyses, we examined the association between the five weight-change patterns across the three key life stages and MASLD. These included weight change from young adulthood (age 25 years) to midlife (ten years before baseline), weight change from midlife to late adulthood (ten years before baseline to one year before baseline, ten years before baseline to baseline, and one year before baseline to baseline), and weight change spanning the entire adult period (age 25 years to baseline). We conducted stratified analyses to evaluate potential effect modification by sex and baseline age (categorised as <60 *vs.* ≥60 years), incorporating interaction terms to formally test for heterogeneity in the observed associations.

We used SAS version 9.4 (SAS Institute Inc., Cary, North Carolina, USA) for all analyses. Statistical significance was defined as two-tailed *P* < 0.05.

## RESULTS

### Characteristics of the study populations

Among 4999 eligible participants, the distribution comprised 2752 (55.05%) in the non-MASLD group, 779 (15.58%) in the MASLD group, and 1468 (29.37%) in the severe steatosis group (**Table 1**). The average age of participants was similar among non-MASLD (mean (x̄) = 58.40; standard deviation (SD) = 13.65), MASLD (x̄ = 58.72; SD = 12.76), and severe steatosis group (x̄ = 57.83; SD = 12.11). The proportion of female participants was higher in the non-MASLD (n = 1630; 59.23%) and MASLD (n = 457; 58.66%) groups than in the severe steatosis group (n = 695; 47.34%). The prevalence of moderate physical activity was similar across groups, with approximately 40% of participants reporting such activity. The severe steatosis group had a slightly higher proportion of smokers (42.64%) than the non-MASLD (38.15%) and MASLD (34.40%) groups did. The total calcium, total cholesterol, aspartate aminotransferase, and alkaline phosphatase were comparable across the three groups. However, the waist circumference and BMI measurements at the four time points (*i.e.* at age 25, ten years prior to baseline, one year prior to baseline, and at baseline) were highest in the severe steatosis group.

**Table 1:** Characteristics of the study population by steatosis severity

	Non-MASLD* (n = 2752)	MASLD* (n = 779)	Severe steatosis* (n = 1468)	*P*-value
**Age in years†**	58.40 (13.65)	58.72 (12.76)	57.83 (12.11)	0.6006
**Gender‡**				<0.0001
Men	1122 (40.77)	322 (41.34)	773 (52.66)	
Women	1630 (59.23)	457 (58.66)	695 (47.34)	
**Education level in years‡**				<0.0001
<9	2228 (80.96)	592 (75.99)	1102 (75.07)	
9–11	440 (15.99)	129 (16.56)	249 (16.96)	
>11	84 (3.05)	58 (7.45)	117 (7.97)	
**Moderate-intensity activity‡**				0.1534
Yes	1044 (37.94)	320 (41.08)	611 (41.62)	
No	1708 (62.06)	459 (58.92)	857 (58.38)	
**Smoked at least 100 cigarettes‡**				0.0076
Yes	1050 (38.15)	268 (34.40)	626 (42.64)	
No	1702 (61.85)	511 (65.60)	842 (57.36)	
**Place of residence‡**				<0.0001
Rural	1816 (65.99)	468 (60.08)	878 (59.81)	
Urban	936 (34.01)	311 (39.92)	590 (40.19)	
**Waist circumference in cm†**	96.43 (14.24)	104.44 (13.52)	112.68 (15.53)	0.0002
**Total calcium in mg/dL†**	9.27 (0.39)	9.29 (0.40)	9.27 (0.39)	0.8038
**Total cholesterol in mmol/L†**	4.93 (1.07)	5.00 (1.07)	4.88 (1.07)	0.9983
**HDL-cholesterol in mmol/L†**	1.49 (0.42)	1.38 (0.41)	1.25 (0.37)	0.0035
**ALT in IU/L†**	18.45 (11.58)	21.59 (13.35)	26.26 (17.60)	<0.0001
**AST in IU/L†**	20.48 (10.14)	21.45 (9.73)	22.77 (14.19)	0.0703
**ALP in IU/L†**	78.00 (27.60)	79.10 (22.34)	82.29 (25.17)	0.1149
**BMI in kg/m^2^†**				
At age 25	24.10 (4.70)	24.77 (5.39)	26.01 (5.84)	<0.0001
Ten years prior to baseline	27.52 (6.41)	29.47 (6.88)	31.40 (7.47)	<0.0001
One year prior to baseline	28.36 (6.69)	31.11 (6.82)	34.00 (7.76)	<0.0001
At baseline	27.91 (6.36)	31.21 (6.64)	34.33 (7.43)	<0.0001
**MD CAP in dB/m†**	224.06 (35.14)	287.12 (8.03)	341.49 (29.11)	<0.0001

ALT – alanine aminotransferase, ALP – alkaline phosphatase, AST – aspartate aminotransferase, BMI – body mass index, CAP – controlled attenuation parameter, HDL – high-density lipoprotein, MASLD – metabolic dysfunction-associated steatotic liver disease, MD – median, SD – standard deviation, x̄ – mean

*Non-MASLD is defined as median CAP<274 dB/m, MASLD as CAP = 274–302 dB/m, and severe steatosis as CAP≥302 dB/m.

†Values are presented as mean (standard deviation).

‡Values are presented as numbers (%).

### Weight change patterns

There was an average increase in BMI of 4.19 kg/m^2^ (SD = 5.16) between 25 years and ten years prior to baseline, with subsequent changes of 1.48 kg/m^2^ (SD = 4.79) from ten years to one year before baseline (Table S2 in the **Online Supplementary Document**). The cumulative BMI change from age 25 to baseline was 5.54 kg/m^2^ (SD = 6.47). More recent intervals showed changes of 1.35 kg/m^2^ (SD = 5.5) from ten years before baseline to baseline, and −0.14 kg/m^2^ (SD = 3.17) during the final year before baseline.

**Figure 2:**
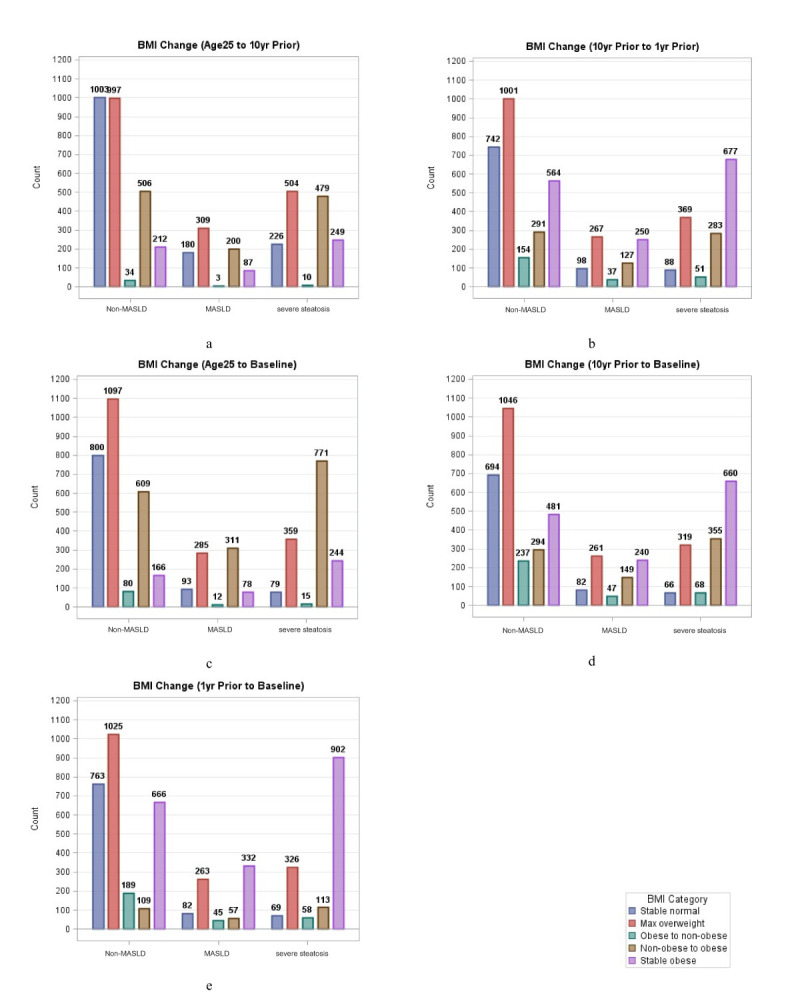
Distribution of BMI change patterns across adulthood by steatosis severity. **Panel A.** BMI Change (Age25 to 10yr Prior). **Panel B.** BMI Change (10yr Prior to 1yr Prior). **Panel C.** BMI Change (Age25 to Baseline). **Panel D.** BMI Change (10yr Prior to Baseline). **Panel E.** BMI Change (1yr Prior to Baseline). BMI – body mass index, MASLD – metabolic dysfunction-associated steatotic liver disease, yr – year.

The weight change patterns displayed distinct distributions across time periods (**Figure 2**, Panels A–E; Table S2 in the **Online Supplementary Document**). The interval from age 25 to ten years prior to baseline included 28.19% stable normal-weight individuals, 9.58% non-obese-to-obese transition, and 10.96% stable obese individuals. In the interval from ten years to one year prior to baseline, there were 18.56% stable normal, 14.02% non-obese to obese, and 29.8% stable obese individuals. From age 25 to baseline, there were 19.44% stable normal, 33.83% non-obese to obese, and 9.76% stable obese individuals, and 16.84% stable normal, 15.96% non-obese to obese, and 27.63% stable obese individuals in the interval of ten years before baseline to baseline.

Distributional differences emerged across steatosis groups, with non-MASLD participants having a higher proportion of stable normal-weight individuals, whereas the severe steatosis group had the highest rate of transition from non-obese to obese status. The severe steatosis group also exhibited the highest maximum prevalence of overweight across all BMI change intervals.

**Figure 3:**
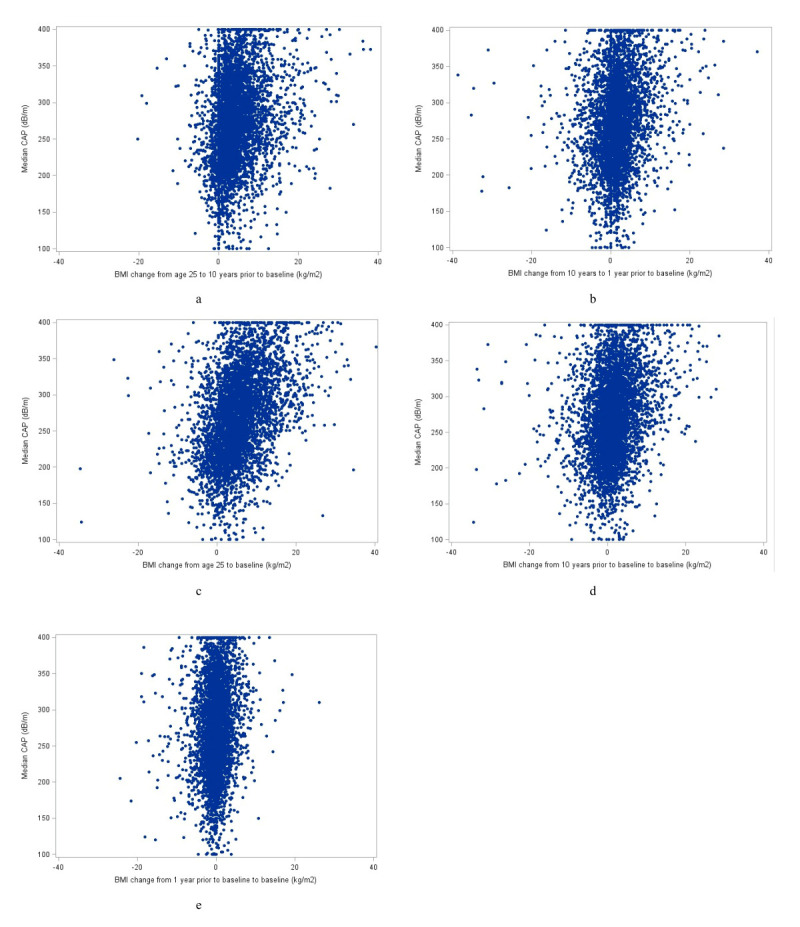
Scatter plots of BMI absolute differences across time intervals and CA*P* values. **Panel A.** BMI change from age 25 to 10 years prior to baseline (kg/m2). **Panel B.** BMI change from 10 years to 1 year prior to baseline (kg/m2). **Panel C.** BMI change from age 25 to baseline (kg/m2). **Panel D.** BMI change from 10 years prior to baseline to baseline (kg/m2). **Panel E.** BMI change from 1 year prior to baseline to baseline (kg/m2). BMI – body mass index, CAP – controlled attenuation parameter.

Consecutive BMI measurements were strongly correlated (r range = 0.68–0.91), with the highest correlation observed between BMI at one year prior to baseline and baseline BMI (r = 0.91). The BMI change measures showed moderate correlations with adjacent change intervals, particularly between BMI change from ten to one year prior and from ten years prior to baseline (r = 0.82) (Figure S1 in the **Online Supplementary Document**). In contrast, correlations between baseline BMI and earlier BMI change intervals were generally weak and negative, indicating that historical weight change captures partially distinct information beyond contemporaneous BMI (**Figure 3**, Panels A–E). All correlation coefficients were statistically significant (*P* < 0.01).

### Associations of weight change patterns with MASLD

Baseline BMI showed the strongest associations, with severe obesity (BMI≥35 kg/m^2^) demonstrating an adjusted OR (aOR) of 3.29 (95% CI = 2.38–4.56) for combined MASLD/severe steatosis and 4.12 (95% CI = 2.70–6.29) for severe steatosis alone. Earlier BMI measurements showed progressively weaker associations, with BMI of ≥35 kg/m^2^ at age 25 years exhibiting non-significant effects (aOR = 0.92; 95% CI = 0.67–1.27) (Table S3 in the **Online Supplementary Document**).

Participants maintaining stable obesity showed significantly increased risks of MASLD or severe steatosis across all examined time intervals (**Table 2**), with strong associations observed from ten years to one year before baseline (OR = 7.89; 95% CI = 6.48–9.61), from age 25 to baseline (OR = 13.19; 95% CI = 10.27–16.94), from ten years before baseline to baseline (OR = 11.27; 95% CI = 9.09–13.98), and from one year before baseline to baseline (OR = 12.13; 95% CI = 9.87–14.90). While these associations remained statistically significant after full adjustment, the effect sizes were substantially attenuated for all time intervals: ten years to one year before baseline (aOR = 1.33; 95% CI = 1.03–1.72), from age 25 to baseline (aOR = 2.07; 95% CI = 1.48–2.90), from ten years before baseline to baseline (aOR = 1.82; 95% CI = 1.36–2.44), and from one year before baseline to baseline (aOR = 2.17; 95% CI = 1.64–2.86). Similar patterns emerged when examining MASLD and severe steatosis risks separately, with aORs for severe steatosis (aOR range = 1.45–2.52) consistently higher than those for the risks of MASLD or severe steatosis and MASLD alone.

**Table 2:** MASLD and severe steatosis with BMI change across adulthood*

	MASLD or severe steatosis		MASLD		Severe steatosis	
	**Model 1**	**Model 2**	**Model 1**	**Model 2**	**Model 1**	**Model 2**
**Age 25 to ten years before baseline**						
Stable normal	ref	ref	ref	ref	ref	ref
Maximum overweight	2 (1.72–2.32)	1.05 (0.89–1.23)	1.77 (1.43–2.18)	1.22 (0.98–1.53)	2.25 (1.87–2.71)	1.10 (0.89–1.37)
Obese to non-obese	1.03 (0.54–1.94)	0.55 (0.27–1.14)	0.50 (0.15–1.65)	0.35 (0.10–1.20)	1.27 (0.61–2.65)	0.67 (0.28–1.64)
Non-obese to obese	3.61 (3.07–4.26)	0.97 (0.79–1.18)	2.31 (1.81–2.93)	1.09 (0.83–1.44)	4.40 (3.59–5.39)	0.99 (0.76–1.27)
Stable obese	4.51 (3.68–5.52)	0.97 (0.76–1.25)	2.53 (1.87–3.44)	1.06 (0.74–1.52)	5.61 (4.4–7.15)	0.93 (0.67–1.28)
**Ten to one year before baseline**						
Stable normal	ref	ref	ref	ref	ref	ref
Maximum overweight	2.51 (2.07–3.04)	1.26 (1.02–1.55)	2.05 (1.58–2.64)	1.36 (1.03–1.78)	3.03 (2.34–3.91)	1.40 (1.06–1.87)
Obese to non-obese	2.36 (1.73–3.22)	1.07 (0.77–1.49)	1.87 (1.22–2.85)	1.2 (0.77–1.88)	2.81 (1.89–4.18)	1.19 (0.77–1.84)
Non-obese to obese	6.45 (5.18–8.04)	1.54 (1.19–2.00)	3.56 (2.63–4.82)	1.59 (1.13–2.25)	9.30 (7.00–12.36)	1.81 (1.29–2.55)
Stable obese	7.89 (6.48–9.61)	1.33 (1.03–1.72)	3.69 (2.82–4.84)	1.37 (0.97–1.94)	11.25 (8.68–14.56)	1.45 (1.03–2.05)
**Age 25 to baseline**						
Stable normal	ref	ref	ref	ref	ref	ref
Maximum overweight	2.70 (2.22–3.28)	1.36 (1.10–1.68)	2.28 (1.76–2.94)	1.57 (1.19–2.07)	3.23 (2.48–4.22)	1.50 (1.11–2.01)
Obese to non-obese	1.71 (1.07–2.73)	1.14 (0.70–1.85)	1.38 (0.72–2.65)	1.16 (0.60–2.26)	1.94 (1.06–3.58)	1.25 (0.65–2.38)
Non-obese to obese	10.27 (8.42–12.52)	2.20 (1.70–2.86)	4.99 (3.82–6.50)	2.21 (1.57–3.13)	15.85 (12.13–20.7)	2.69 (1.90–3.81)
Stable obese	13.19 (10.27–16.94)	2.07 (1.48–2.90)	5.08 (3.54–7.27)	1.95 (1.21–3.12)	19.96 (14.46–27.55)	2.35 (1.51–3.66)
**Ten years before baseline to baseline**						
Stable normal	ref	ref	ref	ref	ref	ref
Maximum overweight	2.55 (2.07–3.14)	1.26 (1.01–1.58)	2.13 (1.62–2.79)	1.43 (1.07–1.92)	3.14 (2.35–4.19)	1.42 (1.04–1.95)
Obese to non-obese	2.31 (1.72–3.08)	1.10 (0.80–1.50)	1.69 (1.14–2.52)	1.16 (0.76–1.77)	2.92 (1.99–4.27)	1.29 (0.85–1.96)
Non-obese to obese	9.96 (7.91–12.55)	2.22 (1.68–2.94)	4.89 (3.58–6.69)	2.14 (1.47–3.12)	16.43 (12.05–22.41)	2.94 (2.01–4.28)
Stable obese	11.27 (9.09–13.98)	1.82 (1.36–2.44)	4.86 (3.64–6.49)	1.84 (1.24–2.71)	17.65 (13.18–23.65)	2.16 (1.46–3.20)
**One year before baseline to baseline**						
Stable normal	ref	ref	ref	ref	ref	ref
Maximum overweight	2.85 (2.32–3.50)	1.40 (1.12–1.75)	2.43 (1.85–3.19)	1.64 (1.23–2.20)	3.39 (2.56–4.50)	1.51 (1.11–2.05)
Obese to non-obese	2.82 (2.09–3.81)	1.23 (0.89–1.70)	2.28 (1.52–3.41)	1.52 (0.99–2.33)	3.27 (2.21–4.86)	1.32 (0.86–2.03)
Non-obese to obese	9.32 (6.98–12.46)	2.51 (1.81–3.48)	5.54 (3.70–8.28)	2.70 (1.72–4.23)	14.32 (9.85–20.84)	3.18 (2.07–4.88)
Stable obese	12.13 (9.87–14.9)	2.17 (1.64–2.86)	5.39 (4.10–7.10)	2.22 (1.53–3.22)	18.57 (14.05–24.55)	2.52 (1.74–3.66)

MASLD – metabolic dysfunction-associated steatotic liver disease, ref – reference

*Values are presented as odds ratios (95% confidence intervals). Model 1 was adjusted for age, gender, race/ethnicity, and survey years, while Model 2 was adjusted for age, gender, race/ethnicity, educational level, family income-to-poverty ratio, moderate activities, smoking status, high-density lipoprotein cholesterol, alanine aminotransferase, aspartate aminotransferase, alkaline phosphatase, waist circumference, and survey years.

Weight transition from non-obese to obese status was significantly associated with MASLD risk, with effect sizes varying by time interval. The transition occurring between ten years and one year before baseline showed a 54% increased risk (aOR = 1.54; 95% CI = 1.19–2.00), whereas stronger associations were observed for longer durations: age 25 to baseline (aOR = 2.20; 95% CI = 1.70–2.86), ten years before baseline to baseline (aOR = 2.22; 95% CI = 1.68–2.94), and one year before baseline to baseline (aOR = 2.51; 95% CI = 1.81–3.48). Both MASLD alone (aOR range = 1.59–2.70) and severe steatosis (aOR range = 1.81–3.18) showed comparable risk estimates for stable obesity.

Similarly, the maximum overweight group exhibited consistent but more modest risk elevations across all examined intervals (aOR range = 1.26–1.51). In contrast, the obese to non-obese group showed no significant associations at any time interval, including the period from age 25 to ten years before baseline (all *P*-values >0.05).

In stratified analyses, men and younger participants (<60 years) showed a higher prevalence of obesity progression across all steatosis severity categories (Table S4 in the **Online Supplementary Document**). Following sex- and age-stratified analyses, the results consistently revealed that the associations between weight change trajectories across adulthood and three hepatic outcomes (combined MASLD/severe steatosis, isolated MASLD, and severe steatosis) were more pronounced in male participants and individuals aged <60 years across all outcome categories (**Table 3**; Tables S5 and S6 in the **Online Supplementary Document**). However, interaction analyses revealed significant sex-specific differences only for the stable obese pattern (*P*-interaction <0.05), whereas no significant age-related interactions were observed for any weight change pattern (all *P*-interaction >0.05)

**Table 3:** Associations between BMI change across adulthood and the risk of MASLD or severe steatosis*

	Sex			Age in years		
	**Men†**	**Women†**	***P*-value**	**<60†**	**≥60†**	***P*-value**
**Age 25 to ten years before baseline**						
Stable normal	ref	ref		ref	ref	
Maximum overweight	1.02 (0.79–1.33)	1.06 (0.86–1.32)	0.9040	1.17 (0.94–1.47)	0.88 (0.69–1.13)	0.2508
Obese to non-obese	0.50 (0.17–1.46)	0.61 (0.23–1.61)	0.5220	0.33 (0.12–0.88)	1.21 (0.42–3.45)	0.8033
Non-obese to obese	0.84 (0.61–1.16)	1.04 (0.81–1.35)	0.8625	0.97 (0.73–1.29)	0.89 (0.67–1.18)	0.6527
Stable obese	1.02 (0.69–1.51)	0.91 (0.65–1.27)	0.0551	1.04 (0.75–1.45)	0.85 (0.57–1.27)	0.4859
**Ten to one year before baseline**						
Stable normal	ref	ref		ref	ref	
Maximum overweight	1.24 (0.90–1.72)	1.28 (0.97–1.68)	0.5824	1.29 (0.97–1.73)	1.18 (0.87–1.60)	0.3383
Obese to non-obese	0.74 (0.44–1.25)	1.50 (0.97–2.32)	0.0111	1.18 (0.71–1.94)	0.95 (0.61–1.49)	0.3979
Non-obese to obese	1.54 (1.00–2.37)	1.53 (1.10–2.11)	0.1648	1.76 (1.24–2.50)	1.25 (0.85–1.85)	0.2353
Stable obese	1.33 (0.88–2.01)	1.29 (0.93–1.8)	0.0022	1.40 (0.97–2.03)	1.22 (0.85–1.75)	0.8107
**Age 25 to baseline**						
Stable normal	ref	ref		ref	ref	
Maximum overweight	1.44 (1.04–1.99)	1.34 (1.01–1.79)	0.3518	1.42 (1.05–1.93)	1.28 (0.95–1.72)	0.4698
Obese to non-obese	1.13 (0.58–2.22)	1.13 (0.55–2.32)	0.5344	0.95 (0.47–1.94)	1.37 (0.69–2.70)	0.6650
Non-obese to obese	2.67 (1.74–4.10)	1.89 (1.35–2.63)	0.1165	2.63 (1.82–3.81)	1.85 (1.27–2.69)	0.1912
Stable obese	2.71 (1.6–4.58)	1.65 (1.05–2.58)	0.0590	2.56 (1.60–4.11)	1.62 (0.98–2.66)	0.1779
**Ten years before baseline to baseline**						
Stable normal	ref	ref		ref	ref	
Maximum overweight	1.52 (1.07–2.17)	1.13 (0.84–1.52)	0.3509	1.48 (1.07–2.05)	1.04 (0.76–1.44)	0.9463
Obese to non-obese	0.99 (0.62–1.58)	1.42 (0.93–2.18)	0.0065	1.16 (0.72–1.89)	0.98 (0.64–1.48)	0.8456
Non-obese to obese	2.91 (1.81–4.67)	1.88 (1.32–2.67)	0.0584	2.86 (1.95–4.21)	1.66 (1.09–2.53)	0.3030
Stable obese	2.40 (1.48–3.88)	1.49 (1.02–2.17)	0.0007	2.40 (1.57–3.68)	1.37 (0.91–2.07)	0.1161
**One year before baseline to baseline**						
Stable normal	ref	ref		ref	ref	
Maximum overweight	1.52 (1.07–2.15)	1.36 (1.01–1.83)	0.7881	1.55 (1.12–2.14)	1.28 (0.94–1.74)	0.2257
Obese to non-obese	1.19 (0.73–1.92)	1.35 (0.86–2.11)	0.2406	1.40 (0.86–2.28)	1.06 (0.68–1.65)	0.5162
Non-obese to obese	2.84 (1.61–4.98)	2.29 (1.53–3.43)	0.6198	3.16 (2.02–4.93)	1.97 (1.19–3.24)	0.3727
Stable obese	2.72 (1.71–4.31)	1.82 (1.28–2.60)	0.0028	2.78 (1.87–4.15)	1.72 (1.16–2.55)	0.2426

MASLD – metabolic dysfunction-associated steatotic liver disease, ref – reference

*Models were adjusted for age, gender, race/ethnicity, educational level, family income-to-poverty ratio, moderate activities, smoking status, high-density lipoprotein cholesterol, alanine aminotransferase, aspartate aminotransferase, alkaline phosphatase, waist circumference, and survey years.

†Values are presented as odds ratios (95% confidence intervals)

## DISCUSSION

In this study, we present novel longitudinal evidence showing the relationship between dynamic weight trajectories across adulthood and MASLD risk. We found that sustained obesity and progressive weight gain from non-obese to obese status were consistently associated with elevated MASLD risk, with effect sizes varying by the timing and duration of weight changes. Notably, baseline BMI showed stronger associations than historical BMI measurements, suggesting cumulative metabolic effects of long-term weight patterns. While men and younger adults (<60 years) generally exhibited stronger associations, significant sex-specific effect modification was only observed for stable obesity. These results extend beyond conventional cross-sectional BMI analyses by characterising how dynamic weight fluctuations influence the risk of hepatic steatosis across the lifespan. Importantly, our findings should be interpreted as complementary to, rather than as replacements for, conventional BMI-based risk assessment.

Previous studies have established obesity as a risk factor for MASLD [15,16]. Yuan and colleagues conducted a Mendelian randomisation study using summary-level data from genome-wide association studies that identified obesity as a potential causal factor in MASLD pathogenesis [15]. Similarly, Xie and colleagues [16] systematically evaluated genetically predicted modifiable risk factors and reported consistent associations between obesity and MASLD. However, these studies predominantly relied on single-time point BMI assessments, thereby limiting their ability to capture the dynamic nature of weight change. Using weight change across adulthood, our findings corroborate and substantially expand prior research in several important ways by demonstrating that weight trajectories provide additional prognostic information beyond static BMI measurements.

The application of lifetime BMI assessment for disease risk prediction has been documented in other contexts, including cancer [17], cardiovascular disease [18], biological age [19], and mortality [8,20]. For instance, our prior work demonstrated that cumulative lifetime exposure to excess weight significantly influences colorectal cancer risk, suggesting a greater impact than previously recognised [17]. A comprehensive meta-analysis by Alharbi and colleagues, involving more than 1.2 million participants, similarly found that weight fluctuations were associated with increased mortality risk compared with stable weight [20]. However, no previous study has assessed the association between obesity and MASLD. We extended this paradigm to MASLD risk assessment, revealing that sustained obesity and progressive weight gain are robustly associated with disease risk. These observations align well with established metabolic pathways, as prolonged adiposity is known to promote chronic insulin resistance, systemic inflammation, and dysregulated lipid metabolism, all key drivers of hepatic steatosis development [21].

An important consideration is that while obesity represents a major risk factor for MASLD, a clinically relevant proportion of patients develop MASLD despite having normal [22]. Population-based screening studies have consistently reported that 8–19% of individuals with BMI<25 kg/m^2^ exhibit MASLD [23–25] underscoring the importance of evaluating weight dynamics rather than relying solely on static BMI measurements. These facts underscore the importance of assessing MASLD by accounting for the dynamic nature of weight fluctuations across the lifespan.

The sex and age differences observed in the present study merit particular attention. The stronger associations in men align with well-documented sex disparities in MASLD prevalence and progression, likely reflecting the protective effects of oestrogen in premenopausal women or sex-specific differences in adipose tissue distribution [26]. Contrary to some previous reports [27,28], we did not observe significant age-related attenuation in metabolic risk among older individuals, possibly because we captured long-term weight patterns rather than relying solely on late-life BMI measurements.

These findings have substantial clinical and public health implications. The particularly strong association between weight gain during young to middle adulthood and MASLD risk suggests that this period may represent a critical window for intervention. Primary care providers should consider incorporating weight trajectory assessments into routine health evaluations, with particular attention to patients who show early signs of progressive weight gain. Our results support the incorporation of weight history into MASLD risk prediction models, as patients with identical current BMI but differing weight trajectories may face substantially different risks. Public health initiatives should emphasise obesity prevention in young adulthood rather than focusing solely on weight reduction after metabolic complications emerge. Future research evaluating MASLD interventions should stratify participants by weight history, as treatment responses may differ substantially between individuals with recent and long-standing obesity.

This study has several strengths, including the use of vibration-controlled transient elastography for the objective assessment of steatosis, detailed weight-history data spanning decades, rigorous adjustment for multiple covariates, and the examination of multiple weight-change intervals to identify critical susceptibility periods. However, the limitations of this study must be acknowledged. While self-reported historical weight data have shown reasonable validity in validation studies, some degree of misclassification is inevitable and would likely bias the results toward the null. Cross-sectional assessment of MASLD status precludes causal inferences and necessitates future prospective studies with repeated CAP measurements. Despite extensive covariate adjustment, residual confounding from unmeasured factors, such as detailed dietary patterns or physical activity intensity, remains possible. Furthermore, because the cohort was derived from older adults in selected communities within Central and Eastern China, the generalisability of the findings to other age groups or populations with different sociodemographic or lifestyle backgrounds may be limited.

Several important questions emerge from these findings and warrant further research. Mechanistic studies should examine whether distinct weight trajectories induce unique metabolic or inflammatory signatures that drive the pathogenesis of MASLD. Clinical trials could test whether interventions targeting specific patterns of weight change are more effective than conventional BMI-based approaches in preventing MASLD. Longitudinal studies with serial CAP measurements are needed to clarify how weight trajectories influence disease progression to advanced stages, including non-alcoholic steatohepatitis and fibrosis. The development of personalised risk prediction models that incorporate weight trajectories along with genetic, metabolic, and imaging biomarkers could significantly enhance the early identification of high-risk individuals.

## CONCLUSIONS

We showed that long-term weight trajectories provide critical prognostic information for MASLD risk beyond conventional BMI assessments. The robust association between sustained obesity and progressive weight gain, particularly during young to middle adulthood, underscores the importance of early and sustained weight-management strategies. Our findings advocate for a paradigm shift in MASLD prevention from static BMI measurements to dynamic assessments of weight patterns, which could enable earlier identification of at-risk individuals and more targeted interventions. Future research should focus on the mechanisms linking specific weight trajectories to hepatic steatosis and developing effective strategies to mitigate these risks across the lifespan.

## Additional material


Online Supplementary Document

## References

[1] Lonardo A Byrne CD Caldwell SH Cortez-Pinto H Targher G Global Epidemiology of Nonalcoholic Fatty Liver Disease: Meta-Analytic Assessment of Prevalence, Incidence, and Outcomes. Hepatology. 2016;64:1388–9. 10.1002/hep.28584 27038241

[2] Estes C Anstee QM Arias-Loste MT Bantel H Bellentani S Caballeria J Modeling NAFLD disease burden in China, France, Germany, Italy, Japan, Spain, United Kingdom, and United States for the period 2016-2030. J Hepatol. 2018;69:896–904. 10.1016/j.jhep.2018.05.036 29886156

[3] Chalasani N Younossi Z Lavine JE Charlton M Cusi K Rinella M The diagnosis and management of nonalcoholic fatty liver disease: Practice guidance from the American Association for the Study of Liver Diseases. Hepatology. 2018;67:328–57. 10.1002/hep.29367 28714183

[4] Cotter TG Rinella M Nonalcoholic Fatty Liver Disease 2020: The State of the Disease. Gastroenterology. 2020;158:1851–64. 10.1053/j.gastro.2020.01.052 32061595

[5] Younossi Z Anstee QM Marietti M Hardy T Henry L Eslam M Global burden of NAFLD and NASH: trends, predictions, risk factors and prevention. Nat Rev Gastroenterol Hepatol. 2018;15:11–20. 10.1038/nrgastro.2017.109 28930295

[6] Quek J Chan KE Wong ZY Tan C Tan B Lim WH Global prevalence of non-alcoholic fatty liver disease and non-alcoholic steatohepatitis in the overweight and obese population: a systematic review and meta-analysis. Lancet Gastroenterol Hepatol. 2023;8:20–30. 10.1016/S2468-1253(22)00317-X 36400097

[7] Fan L Zhao S Shi H Zhang S Role of BMI in the relationship between dietary inflammatory index and non-alcoholic fatty liver disease: an intermediary analysis. Scand J Gastroenterol. 2023;58:1159–65. 10.1080/00365521.2023.2213791 37211749

[8] Chen C Ye Y Zhang Y Pan X Pan A Weight change across adulthood in relation to all cause and cause specific mortality: prospective cohort study. BMJ. 2019;367:l5584. 10.1136/bmj.l5584 31619383 PMC6812615

[9] Castera L Friedrich-Rust M Loomba R Noninvasive Assessment of Liver Disease in Patients With Nonalcoholic Fatty Liver Disease. Gastroenterology. 2019;156:1264. 10.1053/j.gastro.2018.12.036 30660725 PMC7505052

[10] von Elm E Altman DG Egger M Pocock SJ Gøtzsche PC Vandenbroucke JP The Strengthening the Reporting of Observational Studies in Epidemiology (STROBE) statement: guidelines for reporting observational studies. J Clin Epidemiol. 2008;61:344–9. 10.1016/j.jclinepi.2007.11.008 18313558

[11] Stokes A Collins JM Grant BF Scamuffa RF Hsiao C Johnston SS Obesity Progression Between Young Adulthood and Midlife and Incident Diabetes: A Retrospective Cohort Study of US Adults. Diabetes Care. 2018;41:1025–31. 10.2337/dc17-2336 29506982 PMC5911788

[12] Eddowes PJ Sasso M Allison M Tsochatzis E Anstee QM Sheridan D Accuracy of FibroScan Controlled Attenuation Parameter and Liver Stiffness Measurement in Assessing Steatosis and Fibrosis in Patients With Nonalcoholic Fatty Liver Disease. Gastroenterology. 2019;156:1717–30. 10.1053/j.gastro.2019.01.042 30689971

[13] Shao W Gong P Wang Q Ding F Shen W Zhang H Association of exposure to multiple volatile organic compounds with ultrasound-defined hepatic steatosis and fibrosis in the adult US population: NHANES 2017-2020. Front Public Health. 2025;12:1437519. 10.3389/fpubh.2024.1437519 39897180 PMC11782259

[14] Xie R Xiao M Li L Ma N Liu M Huang X Association between SII and hepatic steatosis and liver fibrosis: A population-based study. Front Immunol. 2022;13:925690. 10.3389/fimmu.2022.925690 36189280 PMC9520084

[15] Yuan S Chen J Li X Fan R Arsenault B Gill D Lifestyle and metabolic factors for nonalcoholic fatty liver disease: Mendelian randomization study. Eur J Epidemiol. 2022;37:723–33. 10.1007/s10654-022-00868-3 35488966 PMC9329390

[16] Xie J Huang H Liu Z Li Y Yu C Xu L The associations between modifiable risk factors and nonalcoholic fatty liver disease: A comprehensive Mendelian randomization study. Hepatology. 2023;77:949–64. 10.1002/hep.32728 35971878

[17] Li X Jansen L Chang-Claude J Hoffmeister M Brenner H Risk of Colorectal Cancer Associated With Lifetime Excess Weight. JAMA Oncol. 2022;8:730–7. 10.1001/jamaoncol.2022.0064 35297997 PMC8931669

[18] Yang Y Song L Wang L Li D Chen S Wu S Effect of body mass index trajectory on lifetime risk of cardiovascular disease in a Chinese population: A cohort study. Nutr Metab Cardiovasc Dis. 2023;33:523–31. 10.1016/j.numecd.2022.11.025 36710107

[19] Cao X Yang G Li X Fu J Mohedaner M Danzengzhuoga Høj Jørgensen TS Weight change across adulthood and accelerated biological aging in middle-aged and older adults. Am J Clin Nutr. 2023;117:1–11. 10.1016/j.ajcnut.2022.10.020 36789928

[20] Alharbi TA Paudel S Gasevic D Ryan J Freak-Poli R Owen AJ The association of weight change and all-cause mortality in older adults: a systematic review and meta-analysis. Age Ageing. 2021;50:697–704. 10.1093/ageing/afaa231 33161429

[21] Kivimäki M Strandberg T Pentti J Nyberg ST Frank P Jokela M Body-mass index and risk of obesity-related complex multimorbidity: an observational multicohort study. Lancet Diabetes Endocrinol. 2022;10:253–63. 10.1016/S2213-8587(22)00033-X 35248171 PMC8938400

[22] Fan JG Kim S Wong VW New trends on obesity and NAFLD in Asia. J Hepatol. 2017;67:862–73. 10.1016/j.jhep.2017.06.003 28642059

[23] Fan JG Zhu J Li X Chen L Li L Dai F Prevalence of and risk factors for fatty liver in a general population of Shanghai, China. J Hepatol. 2005;43:508–14. 10.1016/j.jhep.2005.02.042 16006003

[24] Das K Das K Mukherjee PS Ghosh A Ghosh S Mridha AR Nonobese Population in a Developing Country Has a High Prevalence of Nonalcoholic Fatty Liver and Significant Liver Disease. Hepatology. 2010;51:1593–602. 10.1002/hep.23567 20222092

[25] Younossi ZM Stepanova M Negro F Hallaji S Younossi Y Lam B Nonalcoholic Fatty Liver Disease in Lean Individuals in the United States. Medicine (Baltimore). 2012;91:319–27. 10.1097/MD.0b013e3182779d49 23117851

[26] DiStefano JK NAFLD and NASH in Postmenopausal Women: Implications for Diagnosis and Treatment. Endocrinology. 2020;161:bqaa134. 10.1210/endocr/bqaa134 32776116 PMC7473510

[27] Xing J Guan X Zhang Q Chen S Wu SL Sun X Triglycerides Mediate Body Mass Index and Nonalcoholic Fatty Liver Disease: A Population-Based Study. Obes Facts. 2021;14:190–6. 10.1159/000514848 33780962 PMC8138251

[28] Lu S Xie Q Kuang M Hu C Li X Yang H Lipid metabolism, BMI and the risk of nonalcoholic fatty liver disease in the general population: evidence from a mediation analysis. J Transl Med. 2023;21:192. 10.1186/s12967-023-04047-0 36915168 PMC10012451

