# Supplementary material for: Weight change across adulthood in relation to ultrasound-defined metabolic dysfunction-associated steatotic liver disease: a population-based study
Source: J Glob Health. 2026 Mar 20;16:04075. doi: 10.7189/jogh.16.04075 (PMC13002173; doi:10.7189/jogh.16.04075)
Supplement: Online Supplementary Document [file jogh-16-04075-s001.pdf]

Supplement to: Cui C, Shi J, Hao D, Yi C, Zhang Z, Wu C, Yang Y, Zhang Y, Du J, Xu F, Li X. Weight change across adulthood in relation to ultrasound-defined metabolic dysfunction-associated steatotic liver disease: a population-based study. J Glob Health. 2026;16:04075.

Table S1. STROBE Statement—Checklist of items that should be included in reports of ***cohort studies***

	Item No	Recommendation	Page
**Title and abstract**	1	(*a*) Indicate the study's design with a commonly used term in the title or the abstract	1
		(*b*) Provide in the abstract an informative and balanced summary of what was done and what was found	1, 3
**Introduction**			
Background/rationale	2	Explain the scientific background and rationale for the investigation being reported	5
Objectives	3	State specific objectives, including any prespecified hypotheses	5-6
**Methods**			
Study design	4	Present key elements of study design early in the paper	7
Setting	5	Describe the setting, locations, and relevant dates, including periods of recruitment, exposure, follow-up, and data collection	7
Participants	6	(*a*) Give the eligibility criteria, and the sources and methods of selection of participants. Describe methods of follow-up	7
		(*b*) For matched studies, give matching criteria and number of exposed and unexposed	7
Variables	7	Clearly define all outcomes, exposures, predictors, potential confounders, and effect modifiers. Give diagnostic criteria, if applicable	9-10
Data sources/ measurement	8*	For each variable of interest, give sources of data and details of methods of assessment (measurement). Describe comparability of assessment methods if there is more than one group	9-10
Bias	9	Describe any efforts to address potential sources of bias	9-10
Study size	10	Explain how the study size was arrived at	9-10
Quantitative variables	11	Explain how quantitative variables were handled in the analyses. If applicable, describe which groupings were chosen and why	10-11
Statistical methods	12	(*a*) Describe all statistical methods, including those used to control for confounding	10-11
		(*b*) Describe any methods used to examine subgroups	10-11

		and interactions	
		(*c*) Explain how missing data were addressed	10-11
		(*d*) If applicable, explain how loss to follow-up was addressed	Not applicable
		(*e*) Describe any sensitivity analyses	10-11
**Results**			
Participants	13*	(a) Report numbers of individuals at each stage of study—eg numbers potentially eligible, examined for eligibility, confirmed eligible, included in the study, completing follow-up, and analysed	13
		(b) Give reasons for non-participation at each stage	13
		(c) Consider use of a flow diagram	13
Descriptive data	14*	(a) Give characteristics of study participants (eg demographic, clinical, social) and information on exposures and potential confounders	13
		(b) Indicate number of participants with missing data for each variable of interest	13
		(c) Summarise follow-up time (eg, average and total amount)	13
Outcome data	15*	Report numbers of outcome events or summary measures over time	13-14
Main results	16	(*a*) Give unadjusted estimates and, if applicable, confounder-adjusted estimates and their precision (eg, 95% confidence interval). Make clear which confounders were adjusted for and why they were included	13-14
		(*b*) Report category boundaries when continuous variables were categorized	13-14
		(*c*) If relevant, consider translating estimates of relative risk into absolute risk for a meaningful time period	13-14
Other analyses	17	Report other analyses done—eg analyses of subgroups and interactions, and sensitivity analyses	15
**Discussion**			
Key results	18	Summarise key results with reference to study objectives	18
Limitations	19	Discuss limitations of the study, taking into account sources of potential bias or imprecision. Discuss both direction and magnitude of any potential bias	20
Interpretation	20	Give a cautious overall interpretation of results considering objectives, limitations, multiplicity of analyses, results from similar studies, and other relevant evidence	21
Generalisability	21	Discuss the generalisability (external validity) of the study results	21

Other information			
Funding	22	Give the source of funding and the role of the funders for the present study and, if applicable, for the original study on which the present article is based	24

*Give information separately for exposed and unexposed groups.

Note: An Explanation and Elaboration article discusses each checklist item and gives methodological background and published examples of transparent reporting. The STROBE checklist is best used in conjunction with this article (freely available on the Web sites of PLoS Medicine at http://www.plosmedicine.org/, Annals of Internal Medicine at http://www.annals.org/, and Epidemiology at http://www.epidem.com/). Information on the STROBE Initiative is available at http://www.strobe-statement.org.

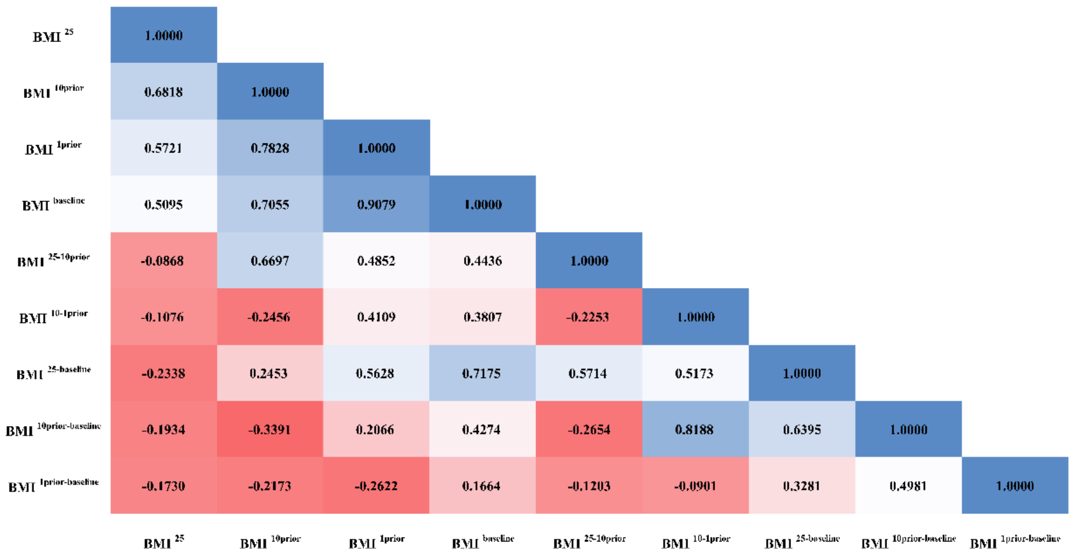

Figure S1. Pairwise Pearson Correlations Between BMI at Four Time Points and Absolute Changes Over Five Intervals

All *P* values <0.01.

BMI, body mass index; BMI 25, BMI at age 25; BMI 10prior, BMI at 10 years prior to baseline; BMI 1prior, BMI at 1 year prior to baseline; BMI baseline, BMI at baseline; BMI 25-10prior, BMI change from age 25 to 10 years prior to baseline; BMI 10-1prior, BMI change from 10 to 1 years prior to baseline; BMI 25-baseline, BMI change from age 25 to baseline; BMI 10prior-baseline, BMI change from 10 years prior to baseline to baseline; BMI 1prior-baseline, BMI change from 1 year prior to baseline to baseline.

Table S2. Distribution of Body Mass Index Change Patterns by Steatosis Severity

	N (%)				Mean±SD (kg/m^2)				
	Non-MASLD	MASLD	Severe steatosis	Overall	Non-MASLD	MASLD	Severe steatosis	Overall	*P*-value
**Age 25 to 10 years before baseline**									
Overall	3246 (100)	921 (100)	1788 (100)	4999 (100)	3.41±4.75	4.71±4.93	5.38±5.72	4.19±5.16	<0.0001
Stable normal	1003 (20.06)	180 (3.6)	226 (4.52)	1409 (28.19)	1.13±1.92	1.94±2.03	1.97±2.09	1.37±1.99	<0.0001
Maximum overweight	997 (19.94)	309 (6.18)	504 (10.08)	1810 (36.21)	2.88±3.03	3.39±2.82	3.47±2.9	3.13±2.97	<0.0001
Obese to non-obese	34 (0.68)	3 (0.06)	10 (0.2)	47 (0.94)	-6.25±3.76	-3.62±0.24	-6.06±3.84	-6.04±3.67	0.2363
Non-obese to obese	506 (10.12)	200 (4)	479 (9.58)	1185 (23.70)	9.19±5.2	9.39±5.2	9.77±6.11	9.46±5.59	0.8086
Stable obese	212 (4.24)	87 (1.74)	249 (4.98)	548 (10.96)	4.48±6.64	4.62±6.7	4.37±6.19	4.45±6.44	0.5881
**10 years before baseline to 1 year before baseline**									
Overall	3246 (100)	921 (100)	1788 (100)	4999 (100)	0.85±4.34	1.64±4.53	2.6±5.49	1.48±4.79	<0.0001
Stable normal	742 (14.84)	98 (1.96)	88 (1.76)	928 (18.56)	0.45±1.64	0.62±1.53	0.88±1.55	0.51±1.62	0.1105
Maximum overweight	1001 (20.02)	267 (5.34)	369 (7.38)	1637 (32.75)	0.71±2.77	1.42±2.31	1.59±2.42	1.03±2.65	<0.0001
Obese to non-obese	154 (3.08)	37 (0.74)	51 (1.02)	242 (4.84)	-5.88±4.59	-6.46±4.16	-5.9±7.05	-5.98±5.13	0.0837
Non-obese to obese	291 (5.82)	127 (2.54)	283 (5.66)	701 (14.02)	5.95±4.05	5.46±3.29	7.03±5.19	6.3±4.47	0.0441
Stable obese	564 (11.28)	250 (5)	677 (13.54)	1491 (29.8)	0.81±5.87	1.53±5.64	2.16±5.67	1.54±5.77	<0.0001
**Age 25 to baseline**									
Overall	3246 (100)	921 (100)	1788 (100)	4999 (100)	3.8±5.78	6.45±6.2	8.32±6.76	5.54±6.47	<0.0001
Stable normal	800 (16.00)	93 (1.86)	79 (1.58)	972 (19.44)	1.05±2.61	2.16±2.39	2.91±1.94	1.31±2.6	0.2704
Maximum overweight	1097 (21.94)	285 (5.7)	359 (7.18)	1741 (34.83)	3.18±3.78	4.51±3.27	4.59±3.1	3.69±3.63	<0.0001
Obese to non-obese	80 (1.6)	12 (0.24)	15 (0.3)	107 (2.14)	-7.25±4.67	-9.23±14.22	-6.57±6.23	-7.37±6.55	<0.0001
Non-obese to obese	609 (12.18)	311 (6.22)	771 (15.42)	1691 (33.83)	9.75±4.92	10.19±4.88	11.48±5.8	10.62±5.39	<0.0001
Stable obese	166 (3.32)	78 (1.56)	244 (4.88)	488 (9.76)	4.68±9.95	6.15±9.32	6.46±8.51	5.8±9.17	<0.0001
**10 years before baseline to baseline**									
Overall	3246 (100)	921 (100)	1788 (100)	4999 (100)	0.39±4.92	1.74±5.39	2.93±6.18	1.35±5.5	<0.0001
Stable normal	694 (13.88)	82 (1.64)	66 (1.32)	842 (16.84)	0.18±2.2	0.34±1.83	1.01±1.81	0.26±2.15	0.3482

Maximum overweight	1046 (20.92)	261 (5.22)	319 (6.38)	1626 (32.52)	0.46±3.2	1.68±2.88	1.59±2.61	0.88±3.09	<0.0001
Obese to non-obese	237 (4.74)	47 (0.94)	68 (1.36)	352 (7.04)	-6.1±4.49	-5.74±7.53	-4.79±4.04	5.8±4.93	<0.0001
Non-obese to obese	294 (5.88)	149 (2.98)	355 (7.1)	798 (15.96)	6.61±4.27	5.98±3.48	7.69±5.03	6.98±4.55	0.0262
Stable obese	481 (9.62)	240 (4.8)	660 (13.2)	1381 (27.63)	0.08±6.59	1.12±6.53	2.01±6.76	1.13±6.72	0.0009
**1 year before baseline to baseline**									
Overall	3246 (100)	921 (100)	1788 (100)	4999 (100)	-0.46±2.98	0.1±3.26	0.34±3.41	-0.14±3.17	<0.0001
Stable normal	763 (15.26)	82 (1.64)	69 (1.38)	914 (18.28)	-0.23±1.54	-0.11±0.99	0.21±1.12	-0.19±1.47	0.0691
Maximum overweight	1025 (20.5)	263 (5.26)	326 (6.52)	1614 (32.29)	-0.16±1.99	0.29±1.71	0.26±1.6	-0±1.88	<0.0001
Obese to non-obese	189 (3.78)	45 (0.9)	58 (1.16)	292 (5.84)	-4.44±3.36	-3.82±6.98	-3.19±2.33	-4.09±4	0.0002
Non-obese to obese	109 (2.18)	57 (1.14)	113 (2.26)	279 (5.58)	3.93±3	3.85±3.32	3.93±3.04	3.91±3.07	0.8142
Stable obese	666 (13.32)	332 (6.64)	902 (18.04)	1900 (38.00)	-0.77±3.91	-0.11±3.07	0.15±3.73	-0.22±3.71	<0.0001

Abbreviations: MASLD, Metabolic dysfunction-associated steatotic liver disease.

Table S3. Odds ratios (95% CIs) of MASLD and Severe Steatosis with BMI at different time points

	OR (95%CI)		
	MASLD or Severe steatosis	MASLD	Severe steatosis
**BMI at Age 25**			
<18.5 kg/m²	1.07 (0.79-1.44)	1.03 (0.69-1.53)	1.04 (0.7-1.54)
18.5-24.9 kg/m²	Reference	Reference	Reference
25-29.9 kg/m²	0.93 (0.80-1.07)	0.93 (0.76-1.14)	0.9 (0.75-1.09)
30-34.9 kg/m²	0.89 (0.70-1.13)	0.94 (0.67-1.33)	0.81 (0.6-1.11)
≥ 35 kg/m²	0.92 (0.67-1.27)	0.62 (0.37-1.04)	0.88 (0.59-1.31)
**BMI at 10 years before baseline**			
<18.5 kg/m²	1.31 (0.68-2.52)	1.10 (0.46-2.66)	1.55 (0.64-3.77)
18.5-24.9 kg/m²	Reference	Reference	Reference
25-29.9 kg/m²	1.09 (0.93-1.29)	1.23 (0.99-1.54)	1.19 (0.96-1.47)
30-34.9 kg/m²	1.06 (0.86-1.29)	1.17 (0.89-1.55)	1.08 (0.83-1.39)
≥ 35 kg/m²	0.93 (0.73-1.17)	0.99 (0.71-1.38)	0.96 (0.71-1.29)
**BMI at 1 year before baseline**			
<18.5 kg/m²	1.18 (0.41-3.37)	0.71 (0.16-3.18)	1.38 (0.33-5.81)
18.5-24.9 kg/m²	Reference	Reference	Reference
25-29.9 kg/m²	1.35 (1.11-1.65)	1.49 (1.15-1.94)	1.49 (1.14-1.95)
30-34.9 kg/m²	1.50 (1.19-1.88)	1.59 (1.17-2.16)	1.66 (1.22-2.24)
≥ 35 kg/m²	1.66 (1.25-2.20)	1.59 (1.08-2.34)	1.87 (1.30-2.70)
**BMI at baseline**			
<18.5 kg/m²	0.35 (0.08-1.50)	0.27 (0.04-1.99)	0.43 (0.06-3.21)
18.5-24.9 kg/m²	Reference	Reference	Reference
25-29.9 kg/m²	1.60 (1.30-1.97)	1.73 (1.32-2.27)	1.81 (1.36-2.40)
30-34.9 kg/m²	2.49 (1.93-3.20)	2.43 (1.73-3.41)	3.06 (2.18-4.28)
≥ 35 kg/m²	3.29 (2.38-4.56)	2.38 (1.52-3.71)	4.12 (2.70-6.29)

Abbreviations: OR, Odds ratios; CI, confidence interval; MASLD, Metabolic dysfunction-associated steatotic liver disease; BMI, body mass index.

Models were adjusted for age, gender, race/ethnicity, educational level, family income-to-poverty ratio, moderate activities, smoking status, waist circumference, high-Density Lipoprotein Cholesterol, Alanine Aminotransferase, Aspartate Aminotransferase, Alkaline Phosphatase, and survey years.

Table S4. Distribution of Body Mass Index Change Patterns by Steatosis Severity, Gender, and Age

	**Non-MASLD (n = 2752, n/%)**		**MASLD (n = 779, n/%)**		**Severe steatosis (n = 1468, n/%)**	
	**Men (n=1122)/Women (n=1630)**	**Age <60 years (n=1432)/ Age ≥60 years (n=1320)**	**Men (n=262) /Women (n=457)**	**Age <60 years (n=400) / Age ≥60 years (n=379)**	**Men (n=773)/Women (n=695)**	**Age <60 years (n=785)/ Age ≥60 years (n=683)**
**Age 25 to 10 years before baseline**						
Stable normal	343 (15.47)/660 (23.72)	649 (24.8)/354 (14.86)	57 (2.57)/123 (4.42)	107 (4.09)/73 (3.06)	99 (4.47)/127 (4.57)	133 (5.08)/93 (3.9)
Maximum overweight	460 (20.75)/537 (19.3)	450 (17.2)/547 (22.96)	165 (7.44)/144 (5.18)	152 (5.81)/157 (6.59)	278 (12.54)/226 (8.12)	274 (10.47)/230 (9.66)
Obese to non-obese	16 (0.72)/18 (0.65)	19 (0.73)/15 (0.63)	1 (0.05)/2 (0.07)	3 (0.11)/0 (0)	5 (0.23)/5 (0.18)	5 (0.19)/5 (0.21)
Non-obese to obese	214 (9.65)/292 (10.5)	181 (6.92)/325 (13.64)	69 (3.11)/131 (4.71)	81 (3.1)/119 (5)	261 (11.77)/218 (7.84)	186 (7.11)/293 (12.3)
Stable obese	89 (4.01)/123 (4.42)	133 (5.08)/79 (3.32)	30 (1.35)/57 (2.05)	57 (2.18)/30 (1.26)	130 (5.86)/119 (4.28)	187 (7.15)/62 (2.6)
**10 years before baseline to 1 year before baseline**						
Stable normal	257 (11.59)/485 (17.43)	447 (17.08)/295 (12.38)	34 (1.53)/64 (2.3)	52 (1.99)/46 (1.93)	39 (1.76)/49 (1.76)	45 (1.72)/43 (1.81)
Maximum overweight	480 (21.65)/521 (18.73)	500 (19.11)/501 (21.03)	142 (6.41)/125 (4.49)	130 (4.97)/137 (5.75)	216 (9.74)/153 (5.5)	186 (7.11)/183 (7.68)
Obese to non-obese	80 (3.61)/74 (2.66)	63 (2.41)/91 (3.82)	16 (0.72)/21 (0.75)	17 (0.65)/20 (0.84)	21 (0.95)/30 (1.08)	21 (0.8)/30 (1.26)
Non-obese to obese	82 (3.7)/209 (7.51)	171 (6.53)/120 (5.04)	47 (2.12)/80 (2.88)	80 (3.06)/47 (1.97)	127 (5.73)/156 (5.61)	181 (6.92)/102 (4.28)
Stable obese	223 (10.06)/341 (12.26)	251 (9.59)/313 (13.14)	83 (3.74)/167 (6)	121 (4.62)/129 (5.42)	370 (16.69)/307 (11.04)	352 (13.45)/325 (13.64)
**Age 25 to baseline**						
Stable normal	296 (13.35)/504 (18.12)	454 (17.35)/346 (14.53)	33 (1.49)/60 (2.16)	42 (1.6)/51 (2.14)	38 (1.71)/41 (1.47)	40 (1.53)/39 (1.64)
Maximum overweight	544 (24.54)/553 (19.88)	529 (20.21)/568 (23.85)	159 (7.17)/126 (4.53)	141 (5.39)/144 (6.05)	214 (9.65)/145 (5.21)	155 (5.92)/204 (8.56)
Obese to non-obese	44 (1.98)/36 (1.29)	45 (1.72)/35 (1.47)	5 (0.23)/7 (0.25)	5 (0.19)/7 (0.29)	10 (0.45)/5 (0.18)	7 (0.27)/8 (0.34)
Non-obese to obese	177 (7.98)/432 (15.53)	297 (11.35)/312 (13.1)	99 (4.47)/212 (7.62)	157 (6)/154 (6.47)	386 (17.41)/385 (13.84)	398 (15.21)/373 (15.66)

Stable obese	61 (2.75)/105 (3.77)	107 (4.09)/59 (2.48)	26 (1.17)/52 (1.87)	55 (2.1)/23 (0.97)	125 (5.64)/119 (4.28)	185 (7.07)/59 (2.48)
**10 years before baseline to baseline**						
Stable normal	257 (11.59)/437 (15.71)	423 (16.16)/271 (11.38)	25 (1.13)/57 (2.05)	40 (1.53)/42 (1.76)	31 (1.4)/35 (1.26)	32 (1.22)/34 (1.43)
Maximum overweight	491 (22.15)/555 (19.95)	508 (19.41)/538 (22.59)	147 (6.63)/114 (4.1)	130 (4.97)/131 (5.5)	195 (8.8)/124 (4.46)	147 (5.62)/172 (7.22)
Obese to non-obese	136 (6.13)/101 (3.63)	97 (3.71)/140 (5.88)	25 (1.13)/22 (0.79)	18 (0.69)/29 (1.22)	36 (1.62)/32 (1.15)	23 (0.88)/45 (1.89)
Non-obese to obese	71 (3.2)/223 (8.02)	187 (7.15)/107 (4.49)	51 (2.3)/98 (3.52)	92 (3.52)/57 (2.39)	156 (7.04)/199 (7.15)	233 (8.9)/122 (5.12)
Stable obese	167 (7.53)/314 (11.29)	217 (8.29)/264 (11.08)	74 (3.34)/166 (5.97)	120 (4.59)/120 (5.04)	355 (16.01)/305 (10.96)	350 (13.37)/310 (13.01)
**1 year before baseline to baseline**						
Stable normal	284 (12.81)/479 (17.22)	431 (16.47)/332 (13.94)	30 (1.35)/52 (1.87)	38 (1.45)/44 (1.85)	30 (1.35)/39 (1.4)	32 (1.22)/37 (1.55)
Maximum overweight	503 (22.69)/522 (18.76)	512 (19.56)/513 (21.54)	146 (6.59)/117 (4.21)	129 (4.93)/134 (5.63)	197 (8.89)/129 (4.64)	146 (5.58)/180 (7.56)
Obese to non-obese	97 (4.38)/92 (3.31)	85 (3.25)/104 (4.37)	21 (0.95)/24 (0.86)	21 (0.8)/24 (1.01)	35 (1.58)/23 (0.83)	24 (0.92)/34 (1.43)
Non-obese to obese	30 (1.35)/79 (2.84)	67 (2.56)/42 (1.76)	16 (0.72)/41 (1.47)	32 (1.22)/25 (1.05)	49 (2.21)/64 (2.3)	74 (2.83)/39 (1.64)
Stable obese	208 (9.38)/458 (16.46)	337 (12.88)/329 (13.81)	109 (4.92)/223 (8.02)	180 (6.88)/152 (6.38)	462 (20.84)/440 (15.82)	509 (19.45)/393 (16.5)

Abbreviations: MASLD, Metabolic dysfunction-associated steatotic liver disease.

Table S5. Associations Between Weight Change Patterns Across Adulthood and the Risk of MASLD

	OR (95% CI)				P interaction a	P interaction b
	Men	Women	Age <60 years	Age ≥60 years		
**Age 25 to 10 years before baseline**						
Stable normal	Reference	Reference	Reference	Reference		
Maximum overweight	1.6 (1.11-2.31)	1.06 (0.86-1.32)	0.96 (0.72-1.29)	0.96 (0.72-1.29)	0.1512	0.9921
Obese to non-obese	0.31 (0.04-2.55)	0.61 (0.23-1.61)	0.34 (0.07-1.62)	0.34 (0.07-1.62)	0.9169	0.4881
Non-obese to obese	0.96 (0.60-1.56)	1.04 (0.81-1.35)	1.15 (0.81-1.62)	1.15 (0.81-1.62)	0.3182	0.4994
Stable obese	1.12 (0.61-2.04)	0.91 (0.65-1.27)	0.99 (0.63-1.56)	0.99 (0.63-1.56)	0.9589	0.4442
**10 years before baseline to 1 year before baseline**						
Stable normal	Reference	Reference	Reference	Reference		
Maximum overweight	1.5 (0.97-2.33)	1.28 (0.97-1.68)	1.35 (0.75-2.42)	1.35 (0.75-2.42)	0.1294	0.4334
Obese to non-obese	1.02 (0.51-2.04)	1.5 (0.97-2.32)	1.42 (0.93-2.17)	1.42 (0.93-2.17)	0.2982	0.4507
Non-obese to obese	1.98 (1.08-3.62)	1.53 (1.10-2.11)	1.47 (0.96-2.25)	1.47 (0.96-2.25)	0.1305	0.5460
Stable obese	1.14 (0.63-2.07)	1.29 (0.93-1.80)	1.01 (1.00-1.02)	1.01 (1.00-1.02)	0.0874	0.6658
**Age 25 to baseline**						
Stable normal	Reference	Reference	Reference	Reference		
Maximum overweight	1.97 (1.26-3.08)	1.34 (1.01-1.79)	1.93 (1.26-2.94)	1.93 (1.26-2.94)	0.0964	0.4004
Obese to non-obese	1.02 (0.36-2.88)	1.13 (0.55-2.32)	1.63 (0.90-2.97)	1.63 (0.90-2.97)	0.4238	0.4472
Non-obese to obese	2.69 (1.45-4.97)	1.89 (1.35-2.63)	1.01 (1.00-1.02)	1.01 (1.00-1.02)	0.8675	0.1688
Stable obese	2.42 (1.11-5.27)	1.65 (1.05-2.58)	1.56 (1.00-2.42)	1.56 (1.00-2.42)	0.9437	0.3455
**10 years before baseline to baseline**						
Stable normal	Reference	Reference	Reference	Reference		
Maximum overweight	2.18 (1.33-3.58)	1.13 (0.84-1.52)	1.62 (1.01-2.59)	1.62 (1.01-2.59)	0.0158	0.4697
Obese to non-obese	1.42 (0.74-2.74)	1.42 (0.93-2.18)	1.01 (1.00-1.02)	1.01 (1.00-1.02)	0.4533	0.9320
Non-obese to obese	3.83 (1.92-7.61)	1.88 (1.32-2.67)	1.62 (1.04-2.52)	1.62 (1.04-2.52)	0.0804	0.9844
Stable obese	2.12 (1.05-4.27)	1.49 (1.02-2.17)	1.05 (0.67-1.64)	1.05 (0.67-1.64)	0.1104	0.1031
**1 year before baseline to baseline**						
Stable normal	Reference	Reference	Reference	Reference		
Maximum overweight	1.90 (1.19-3.04)	1.36 (1.01-1.83)	1.01 (1.00-1.02)	1.01 (1.00-1.02)	0.1189	0.9187
Obese to non-obese	1.46 (0.74-2.87)	1.35 (0.86-2.11)	1.53 (0.99-2.38)	1.53 (0.99-2.38)	0.4826	0.5400
Non-obese to obese	2.75 (1.20-6.32)	2.29 (1.53-3.43)	1.00 (0.64-1.57)	1.00 (0.64-1.57)	0.7813	0.7997
Stable obese	2.45 (1.27-4.74)	1.82 (1.28-2.60)	0.78 (0.55-1.12)	0.78 (0.55-1.12)	0.8893	0.1688

Abbreviations: MASLD, Metabolic dysfunction-associated steatotic liver disease; OR, odds ratio; CI, confidence interval.

[a], *P* for interaction between sex.

[b], *P* for interaction between age.

Models were adjusted for age, gender, race/ethnicity, educational level, family income-to-poverty ratio, moderate activities, smoking status, high-Density Lipoprotein Cholesterol, Alanine Aminotransferase, Aspartate Aminotransferase, Alkaline Phosphatase, waist circumference, and survey years.

Text S1. Explanation of authorship change statement

Due to the authors' oversight, a joint authorship indication was omitted for one co-author (DH), while another author (XC) was accidentally designated as a corresponding author instead of FX. These omissions only occurred with the submitted version of the manuscript, while the authorship list entered into the Journal's submission system was initially correct. All authors have signed a form agreeing to these changes and have provided evidence of the authors' initial contributions in the form of meeting minutes and discussion chains.

Table S6. Associations Between Weight Change Patterns Across Adulthood and the Risk of Severe Steatosis

	OR (95% CI)				*P* interaction a	*P* interaction b
	**Men**	**Women**	**Age <60 years**	**Age ≥60 years**		
**Age 25 to 10 years before baseline**						
Stable normal	Reference	Reference	Reference	Reference		
Maximum overweight	0.98 (0.71-1.35)	1.22 (0.91-1.63)	1.22 (0.91-1.63)	1.22 (0.91-1.63)	0.6798	0.1053
Obese to non-obese	0.61 (0.17-2.15)	0.75 (0.22-2.53)	0.75 (0.22-2.53)	0.75 (0.22-2.53)	0.5920	0.2800
Non-obese to obese	0.83 (0.55-1.23)	1.09 (0.78-1.52)	1.09 (0.78-1.52)	1.09 (0.78-1.52)	0.9315	0.5276
Stable obese	0.98 (0.60-1.59)	0.84 (0.55-1.31)	0.84 (0.55-1.31)	0.84 (0.55-1.31)	0.0860	0.1892
**10 years before baseline to 1 year before baseline**						
Stable normal	Reference	Reference	Reference	Reference		
Maximum overweight	1.33 (0.87-2.03)	1.52 (1.03-2.24)	1.84 (1.03-3.3)	1.84 (1.03-3.30)	0.8455	0.3900
Obese to non-obese	0.72 (0.37-1.42)	1.84 (1.03-3.30)	1.80 (1.15-2.81)	1.80 (1.15-2.81)	0.0135	0.7409
Non-obese to obese	1.84 (1.06-3.18)	1.8 (1.15-2.81)	1.35 (0.86-2.13)	1.35 (0.86-2.13)	0.2873	0.1906
Stable obese	1.52 (0.90-2.58)	1.35 (0.86-2.13)	1.01 (1.00-1.02)	1.01 (1.00-1.02)	0.0107	0.7574
**Age 25 to baseline**						
Stable normal	Reference	Reference	Reference	Reference		
Maximum overweight	1.54 (1.01-2.35)	1.53 (1.01-2.3)	2.24 (1.41-3.57)	2.24 (1.41-3.57)	0.2045	0.2775
Obese to non-obese	1.37 (0.60-3.15)	1.00 (0.34-2.96)	1.76 (0.96-3.22)	1.76 (0.96-3.22)	0.9728	0.4447
Non-obese to obese	3.47 (2.02-5.98)	2.24 (1.41-3.57)	1.01 (1.00-1.02)	1.01 (1.00-1.02)	0.5996	0.1378
Stable obese	3.42 (1.77-6.59)	1.76 (0.96-3.22)	1.21 (0.8-1.82)	1.21 (0.8-1.82)	0.2793	0.1645
**10 years before baseline to baseline**						
Stable normal	Reference	Reference	Reference	Reference		
Maximum overweight	1.54 (0.98-2.44)	1.36 (0.88-2.12)	1.75 (1.03-2.97)	1.75 (1.03-2.97)	0.6996	0.6119
Obese to non-obese	1.03 (0.57-1.88)	1.82 (1.01-3.28)	1.01 (1.00-1.02)	1.01 (1.00-1.02)	0.0159	0.9793
Non-obese to obese	3.71 (2.04-6.77)	2.55 (1.55-4.19)	1.19 (0.78-1.80)	1.19 (0.78-1.80)	0.2309	0.2038
Stable obese	2.81 (1.53-5.16)	1.75 (1.03-2.97)	0.68 (0.44-1.04)	0.68 (0.44-1.04)	0.0172	0.1723
**1 year before baseline to baseline**						
Stable normal	Reference	Reference	Reference	Reference		
Maximum overweight	1.72 (1.09-2.72)	1.44 (0.94-2.20)	1.01 (1.00-1.02)	1.01 (1.00-1.02)	0.8790	0.1462
Obese to non-obese	1.40 (0.76-2.60)	1.37 (0.73-2.54)	1.21 (0.80-1.83)	1.21 (0.80-1.83)	0.5099	0.7108
Non-obese to obese	3.78 (1.86-7.70)	2.84 (1.65-4.91)	0.70 (0.46-1.07)	0.70 (0.46-1.07)	0.9525	0.3224
Stable obese	3.74 (2.07-6.75)	1.95 (1.20-3.18)	0.58 (0.41-0.81)	0.58 (0.41-0.81)	0.0258	0.2122

Abbreviations: MASLD, Metabolic dysfunction-associated steatotic liver disease; OR, odds ratio; CI, confidence interval.

[a], *P* for interaction between sex.

[b], *P* for interaction between age.

Models were adjusted for age, gender, race/ethnicity, educational level, family income-to-poverty ratio, moderate activities, smoking status, high-Density Lipoprotein Cholesterol, Alanine Aminotransferase, Aspartate Aminotransferase, Alkaline Phosphatase, waist circumference, and survey years.
